# A System for Monitoring of Broadband FMR Phenomenon in Low-Carbon Steel Films Subjected to Deformations

**DOI:** 10.3390/s21134301

**Published:** 2021-06-23

**Authors:** Grzegorz Psuj, Przemyslaw Lopato, Michal Maciusowicz, Michal Herbko

**Affiliations:** Center for Electromagnetic Fields Engineering and High-Frequency Techniques, Faculty of Electrical Engineering, West Pomeranian University of Technology, ul. Sikorskiego 37, 70-313 Szczecin, Poland; plopato@zut.edu.pl (P.L.); michal.maciusowicz@zut.edu.pl (M.M.); michal.herbko@zut.edu.pl (M.H.)

**Keywords:** ferromagnetic resonance, measuring system, coplanar waveguide, low carbon steel film, deformation

## Abstract

Stresses and deformations are some of the main factors influencing the mechanical and magnetic properties of steels. Resonance methods, based on the utilization of high-frequency electromagnetic fields, are the ones that can provide information about the course of the magnetization process. Moreover, according to skin effect, these methods may show sensitivity to surface deformations of the examined materials as well. As a rule, however, they are used to study the properties of materials of very limited sizes. This paper presents an approach in which a system based on the ferromagnetic resonance method FMR was considered for monitoring changes of characteristics related to magnetization dynamics of steel elements subjected to deformations. First, a solution was proposed, and then a numerical analysis, as well as a construction of the system, were presented. During the study, the possibility of carrying out measurements in a wide range of electromagnetic field conditions, enabling local inspection on structures, was also analysed. The system operation was verified using a set of samples made of low carbon steel film, representing distinct states of deformation. The obtained results make it possible to clearly distinguish changes in magnetic conditions, pointing to changes in the resultant magnetic anisotropy caused by the straining process.

## 1. Introduction

The study of the influence of stresses and deformations on the properties of magnetic materials is one of the key issues in modern engineering. One way to get to know often complex relationships is to observe the dynamics of magnetization. This process in magnetic materials is influenced by a number of factors referring to their micro- and macrostructural properties [1,2]. For this reason, observation of features related to the magnetization process enables the characterization and assessment of the state of magnetic structures. Many methods allow one to observe the dynamics of the magnetization process. In macroscopic terms, these methods can refer to, inter alia, the observation of properties based on the hysteresis loop or the Barkhausen effect [1,3,4,5]. However, with regard to micro-scale characterization, methods that use magneto-optical phenomena find their application [6,7]. The latter group also includes methods based on resonance phenomena, which in the case of magnetic materials specifically refers to ferromagnetic resonance FMR [2,8,9,10,11,12,13].

In this method, application of external static (DC) magnetic field *H*_DC_ to magnetic material causes precession of magnetization vector *M*. Because of the damping process, the angle of precession decreases resulting in relaxation characteristics. By application of orthogonal high-frequency magnetic field *H*_RF_ (so-called pumping field) of a specific frequency, the magnetization precession about the equilibrium direction can be maintained. Resonating atoms absorb energy. If the frequency of *H*_RF_ is almost equal to the precessional one, the maximum energy absorption by the ferromagnetic material is achieved. Thus, by sweeping the RF frequency the resonant conditions can be determined. The dynamics are described by the Landau–Lifshitz–Gilbert equation referring to both the precessional and the damping torque of the magnetization [2,7,9,14].

The FMR technique was introduced in the middle of the 20th century and is well known. During the standard FMR experiment, a sample is placed inside a microwave resonator, and a system is under the operation of an excitation wave at a fixed high-frequency [2,8,9,10,11,12,13,14]. By sweeping of an external bias DC field, perpendicular to the excitation one, the resonance conditions are being found. The technique is well established. The utilized resonator cavity present high sensitivity to the FMR phenomenon and the results are relatively straightforward to interpret because of the microwave field uniformity. On the other hand, the measurements are conducted only for a single frequency while the magnetic conditions are varying and thus the amount of provided information can be limited [10,14]. However, due to the technological advancement of electronic instrumentation new possibilities of measurements methodology have been introduced in recent years. Such an example is the pulsed inductive microwave magnetometry PIMM or the vector network analyzer ferromagnetic resonance VNA-FMR [10,11,12,14,15,16,17]. In the case of the VNA-FMR, the high-frequency field is being provided to a system by a stripline (SL) or a coplanar waveguide (CPW) which is placed in the centre of strong electromagnets usually, based on Helmholtz coils unit. The utilization of VNA allows to provide to the CPW a high band wave of a wide frequency range and collect the response of the system while changing the level of the DC external field. This result in achieving of absorption characteristic for any fixed DC magnetic field level, which allows one to study the dynamic response of the magnetization of the tested material in specific conditions. In the case of the PIMM, method the excitation field is provided transversely to the fixed DC magnetic field, in the form of the impulse or step. Then, the response is converted to frequency-domain through the Fast Fourier Transform FFT analysis (requirement of post-processing). In each case, detectable resonances are in the range of GHz. The comparison of the performance of those techniques was provided by various authors. In [10], the VNA-FMR method was studied along with PIMM and conventional FMR for epitaxial ultrathin Fe films. All techniques were then additionally comparted with the time-resolved magneto-optic Kerr effect method. The obtained results confirmed the good agreement between the techniques. However, in the case of inductive ones, the highest signal-to-noise ratio (SNR) was achieved for VNA-FMR one. A similar comparison was presented in [11], where the authors analysed the linewidth for a series of Permalloy films of different thickness obtained by FMR, VNA-FMR and PIMM methods. The shown results confirmed the consistency of the data acquired for a different technique. Nevertheless, the VNA-FMR technique still provided greater flexibility, allowing modifications of both field components. Moreover, the reports confirmed the good reliability of the technic. Thus, considering the possibility of wide control of measuring conditions and all the above-mentioned aspects, the VNA-FMR method was applied in this paper.

In general, the FMR absorption line is influenced by a number of factors related to magnetocrystalline, magnetoelastic or shape anisotropy and demagnetizing field. These factors influence the total magnetic energy and the effective internal field of the material [10,18,19,20]. The shape of the sample and the orientation of the DC magnetic field (in relation to symmetry axes of the material’s crystal) as well as the temperature, all influence the position of the absorption line [9,19,21,22,23,24]. The width of the FMR resonant line is related to the damping of magnetization precession and the uniformity of the sample. Several factors influence the damping of FMR, having an internal and external character. The internal contributions are related to the properties of a given material [2,7,20] (i.e., the effect of eddy current damping), while the external ones are associated with defects in the sample [20,25,26,27]. In polycrystalline materials, the line broadening mechanism can be dominated by magnetocrystalline anisotropy (in conjunction with polycrystalline structure) [19,21,28]. Another key issue affecting the measurements is the size of the tested object. The dimensions and the shape influence the magnetic anisotropy and the demagnetizing field strength [20,29,30,31,32]. The methods based on the FMR phenomenon are mainly used for testing materials, in which at least one dimension is significantly reduced (i.e., thin films or thin wires) [14]. However, also few studies have been carried out to show the possibility of using this method to evaluate the properties of larger-scale elements. In [30], the authors analysed three designs of the inductive probe in the form of the coplanar waveguide or microstrip line for characterization of structures in wafer scale. The obtained damping values showed a tendency to even decrease with the increasing size of the sample, which was explained by the samples’ volume positive impact on SNR of measurements. Similar observations were made in [29].

In the context of the above-mentioned factors, the FMR absorption characteristic can provide information about the state of the material, as deformations in steels significantly affect changes in their structure and properties. Growing strains increase the number of defects in the lattice, which cause the broadening of the absorption line [25,28]. In the case of polycrystals, grain boundaries are an additional influencing element. The grain size and texture being affected by the deformations also play a vital role, as they influence the magnetization [29]. Deformation of the crystal lattice causes anisotropy, which also results in the appearance of directions of easy and hard magnetization axes in the material. Their distribution may also interchange depending on the stage of the deformation process [25]. Additionally, the deformation process itself in the steels strongly affects the properties in surface layers and further the resultant magnetic anisotropy characteristic [1,2,19,25,26]. Thus, although the testing depth for the FMR frequency range is limited by the skin depth effect, the method may be promising for the evaluation of material changes.

Considering all discussed aspects, this paper aims to investigate the possibility of building a measurement system, based on the adaptation of the VNA-FMR technique, enabling effective sensing of the scale of changes obtained for construction steel as a result of its deformation. It should be notated that the assessment of the influence of individual factors is not simple and depends strictly on the tested material and the deformation process conditions. The properties are influenced by the level and the speed of deformation or the environmental temperature [9]. In the light of the above-mentioned properties, the physical interpretation of the observed absorption characteristics is a complex process, requiring detailed analysis, including the evaluation of the mechanical and magnetic microstructure. Therefore, the main purpose of the paper is to carry out a study covering the design process, construction of the system and verification measurements allowing initial evaluation of the scale of the response of the proposed VNA-FMR system. It must be mentioned that the measurements were made on relatively small-scale samples (that is 17 mm × 17 mm). However, the magnetizing unit and the CPW were designed to allow carrying out the measurements also locally on the surface. All measurements were conducted under repeatable conditions. This let the observation of relative changes in FMR characteristics, thanks to which it was possible to minimize the influence of repeating influencing or damping factors on the occurrence of resonance and linewidth. Thus, it allowed to emphasize the changes and to evaluate the impact of the deformation of the tested material.

## 2. VNA Based FMR Measuring System

**Figure 1:**
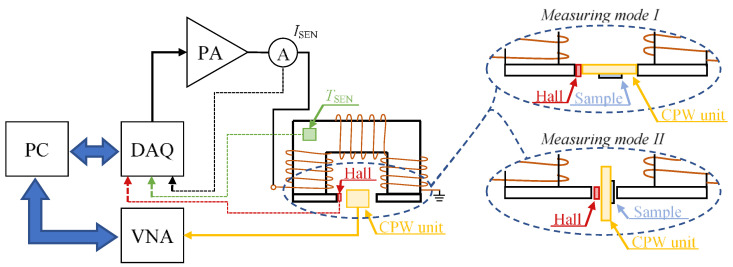
Scheme of the measuring system and visualization of both CPW measuring modes.

The idea of the proposed system was to make it possible to inspect the sample locally. Therefore the mobility of the whole system was an important aspect of the construction. The scheme of the measurement system is shown in Figure 1. The system consists of two sections: magnetizing and measuring. The main element of the first section is the unit, which task is to generate the DC field magnetizing of the tested material. The second section is based on the use of the VNA along with the CPW to observe the phenomenon of ferromagnetic resonance. Detailed system configuration will be described in the following sections.

### 2.1. DC Field Magnetization/Demagnetization Section

**Figure 2:**
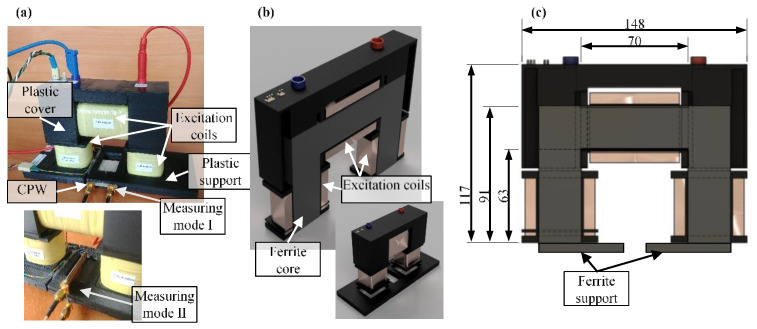
DC field magnetization unit: (**a**) photo of unit in both magnetizing modes, (**b**) 3D visualization with cross-section, (**c**) 2D cross-section with dimensions; all dimensions are given in mm.

The magnetization section aims to generate a static (DC) magnetic field and a demagnetizing magnetic pulse as well (to adjust initial conditions each time before the measurements). The designed solution is based on the use of a U-shaped ferromagnetic core, on which three sections of the excitation windings were fixed having a total number of turns N equal to 1765. Additionally, to drive and concentrate the magnetic flux, enabling it to reach a higher value of the magnetizing field in the tested material, the ends of both columns of the ferrite core can be fitted with additional ferrite supports. This can be especially beneficial in case of structures of size smaller than the distance between the columns. This allowed one to adjust the distance between the columns defining an air path length depending on the given operating mode of the measuring system (see Figure 1). The shape and dimensions of the magnetizing unit are shown in Figure 2. The winding is made of a wire having a diameter of 0.65 mm, meeting the IEC 317-13 standard and allowing operation at temperatures even up to 200 °C. The windings themselves were wound on carcasses designed to optimally use the available space. Then, they were manufactured using 3D printing technology from ABS plastic resistive to an increased operating temperature (exceeding 100 °C). The developed design of the magnetizing unit makes it possible to carry out measurements on the surface of the tested materials. It allows one to move over the tested element which dimensions are not limited by the system ones. During the conceptualization, the stated system’s mobility requirements affected the need to miniaturize the whole structure. In consequence, considering high currents flow in the magnetizing head, the temperature influence on the unit became a valid aspect. This makes the resistance of individual elements to possible high operating temperatures a crucial factor during the prototyping.

The operation of the system was managed by a personal computer equipped with a data acquisition card (DAQ) containing both an analogue-to-digital (A/D) and digital-to-analogue (D/A) converters. The magnetizing signal shape was first introduced by the D/A card, and then amplified in a power amplifier (PA) and fed to the excitation coils. During the measurements, both the current provided to the magnetizing system and the value of the magnetic field in the close vicinity of the high-frequency unit (the CPW with the tested material) were controlled by the Hall sensor element. Due to the clear dependence of the magnetic parameters of the core on the temperature, the system was additionally equipped with a temperature sensor. This allowed us to appropriate control the data acquisition procedure. The data from the sensors were recorded using the A/C converter of the DAQ card. To create repeatable measurement conditions, each time before starting the measurements, the system was demagnetized. For that need, the demagnetizing pulse was generated, amplified and then driven to the input of the excitation coils.

### 2.2. High-Frequency Electromagnetic Field Sensing Section

The measuring section consists of two elements: the VNA and the specially designed and made CPW. The used VNA (pocketVNA [33]) is a small-sized solution powered by a USB port, which enables its full integration with the system and supports high mobility requirements. The used VNA allows one to obtain the dynamic range of the system at the level of 70 dB for 350 MHz and 40 dB for 4 GHz, which is sufficient for the implementation of the system. The key element of the system is the second element of the measuring section–the CPW waveguide.

**Figure 3:**
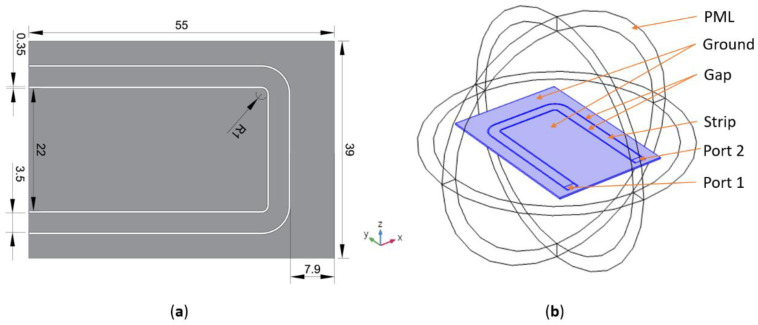
Coplanar waveguide: (**a**) dimensions (in mm) of the designed CPW, (**b**) the FEM model (the active part of CPW is parallel to the *x*-axis).

**Figure 4:**
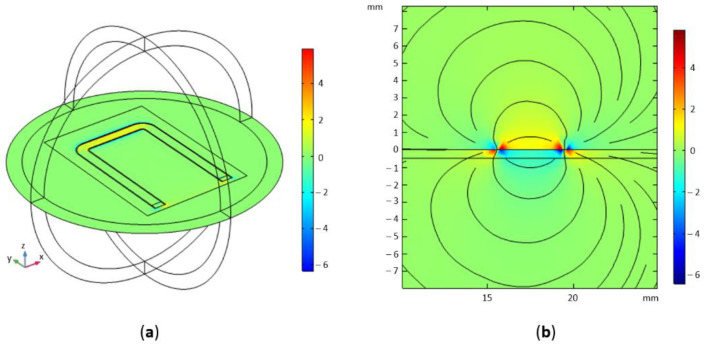
Results of FEM simulation—magnetic field *H*_RFy_ distribution [A/m] for *f* = 2 GHz: (**a**) on the surface of CPW, (**b**) in cross-section (*y-z* plane); assumed excitation power on port 1 equals to 1 W.

**Figure 5:**
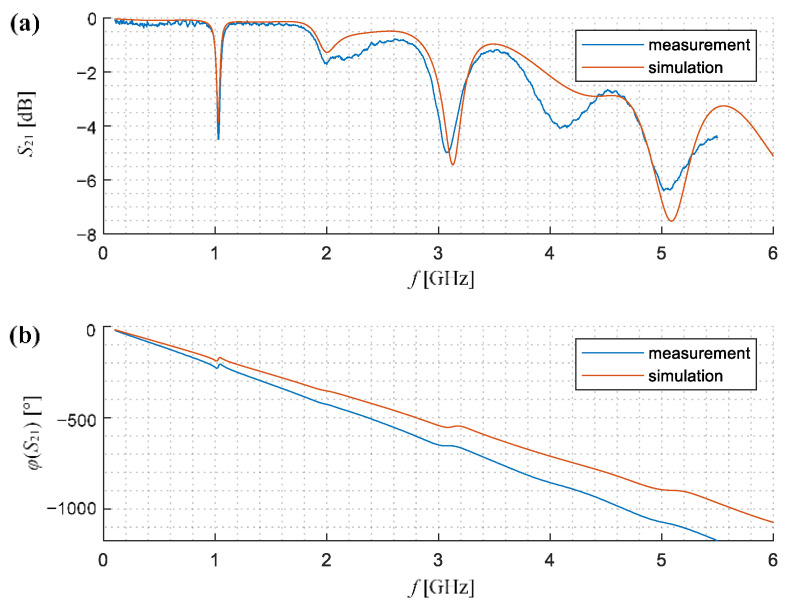
Comparison of the measured and the simulated scattering parameter *S*_21_ characteristic obtained for the designed CPW: (**a**) amplitude |*S*_21_|, (**b**) phase *φ*(*S*_21_).

The coplanar waveguide is a part of the FMR system that is responsible for the injection of high-frequency magnetic field component *H*_RF_ perpendicular to the DC component of magnetic field *H*_DC_. Conventional CPW (utilized in this work) consists of a central conductor and two side conductive areas connected to the ground in contrast to conductor-backed coplanar waveguide (CBCPW), where there is an additional ground plane. The electromagnetic wave propagating through CPW is a quasi-TEM wave [34]. The transmission line was designed on Rogers RO4003c material because of its stability in both frequency and temperature. Contrary to both conductive centre strip and dielectric gap width, the laminate thickness *h*_s_ is not a crucial feature (its influence on magnetic field distribution in the cross-section of CPW is relatively low). Thus the *h*_s_ = 0.508 mm was chosen because it enables to achieve enough mechanical stiffness of sensing element. The average dielectric constant of the utilized laminate was *ε*_r_ = 3.38. The calculations were carried out in a commercial, finite element method (FEM) based Comsol Multiphysics environment (Figure 3 and Figure 4). The dimensions of the designed CPW are illustrated in Figure 3a. The geometry of the numerical model is shown in Figure 3b. The active part of the CPW (parallel to the *x*-axis depicted in Figure 3b) is driven through port 1 and parallel to the *y* axis transmission line sections, and the detection is performed in port 2 (scattering matrix parameter *S*_21_ is calculated). In the physical implementation of the CPW, the SMA connectors were utilized at both ends of the transmission line. To simplify the numerical model, planar lumped ports were used, as was recommended in [35]. This simplification is valid and do not cause noticeable error—it was verified using measurements of unloaded CPW shown in the further part of this subsection (Figure 5).

The Magnetic field *H*_RFy_ distribution for *f* = 2 GHz is shown in Figure 4. As expected the magnetic field *y*-component is distributed in the vicinity of CPW with a high concentration close to the CPW gap. In the area of increased concentration, the strength of the magnetic field is approximately five times higher. The obtained *H*_RFy_ distribution enables injection of the magnetic field to the steel films separated by a thin (order of 10–300 μm) dielectric (isolating) layer.

To verify the quality of the performance of the manufactured CPW waveguide, the measurements of the characteristics in the range from 100 MHz to 5.5 GHz were carried out. Then, their results were compared with the results obtained during numerical simulations. Figure 5 presents the course of the amplitude and the phase of the *S*_21_ transmission coefficient of the scattering matrix received for both empirical and numerical experiments. Especially in the case of the amplitude, a large convergence of both characteristics was obtained (a calculated cross-correlation reach out 0.96 and the standard deviation between them is equal to 0.29 dB). Simultaneously one can notice that the differences between both waveforms (the measured and the computed ones) clearly increase with the frequency. Taking into account the above factors and the ability to work in the range with relatively high system dynamics, the final measurements were carried out in the frequency range from 100 MHz to 4 GHz for 1201 of frequency points.

## 3. Experiment

The actual experiment was performed for a set of deformed samples made of low-carbon steel film. During the measurements, two modes of orientation setting of the CPW in relation to the direction of the static component of the magnetic field *H*_DC_ were taken into account: the first, in which the CPW plane was parallel, and the second, in which the CPW plane was perpendicular to the direction of the *H*_DC_ component. In both cases, the CPW was arranged in such a way that the *H*_DC_ component of the field was perpendicular to the high-frequency component of the pumping field *H*_RF_. Details of the test samples and measurements are provided in the following subsections.

### 3.1. Measuring Samples

All samples for which the tests were carried out were made of 100 μm thick film made of low carbon steel DC04 referring to the EN 10139 standards (grade 1.0338). The chemical composition and basic mechanical parameters of the steel are presented in Table 1 and Table 2.

**Figure 6:**
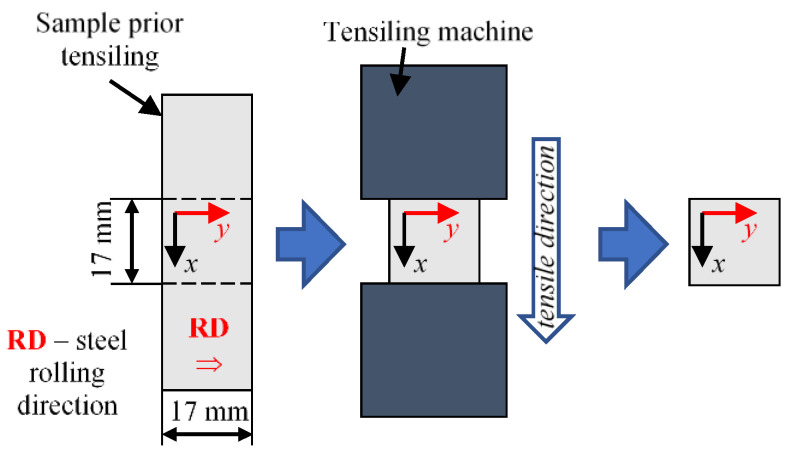
Measuring samples preparation and straining procedure (from **left** to **right**): initial cutting out the samples prior to straining, tensile loading and final cutting to desired dimensions.

A set of five samples was prepared for the experiments. The samples straining procedure is shown in Figure 6. At the first stage, material strips were cut from the film sheet. All were cut transversely to the rolling direction of the steel, i.e., so that the shorter dimension of the film strips (indicated by the *y*-direction) was aligned along the rolling direction. In the next step, the strips were strained using a hydraulic machine and then unloaded. At the last stage, the middle (active) parts of the samples, that were placed between the jaws of the loading machine, were cut out from all strips. Finally, square samples of steel film (17 mm × 17 mm) were obtained. Such shape allowed to carry out measurements in convergent conditions for both directions of the samples (symmetric shape under the terms of both directions). Based on the optical evaluation, the loading process finally provided: two samples (*s*_B_ and s_C_) with a deformation of (0.3 ± 0.1)% and two (*s*_D_ and *s*_E_) with a deformation of (0.5 ± 0.1)%. Additionally, a reference sample was also prepared that was not strained (sample *s*_A_). As a result, that allowed us to observe the general changes in the material properties. Then, the FMR measurements were made ex-situ.

### 3.2. Measurements, Results and Discussion

Final measurements were performed in two stages. The first one aimed to determine the influence of the CPW orientation mode on the possibility of observing the FMR phenomenon. For this purpose, first, the measurements were made in both setting modes (I and II) for different levels of the magnetizing current for the reference *s*_A_ sample. For both modes, the value of the relative complex gain factor was calculated as *G*_ref_ = *S*_21_/*S*_21(0)_, where *S*_21_ was the value of the complex scattering factor for a given DC magnetizing current *I* and frequency of *H*_RF_ and *S*_21(0)_ was the value of the scattering factor for a given frequency of *H*_RF_ and the current of 0 A (no static field). On this basis, the scalar value of the relative gain factor defined in a logarithmic scale was determined:

*g*_ref_ = 20⋅log_10_ (|*G*_ref_|). (1)

**Figure 7:**
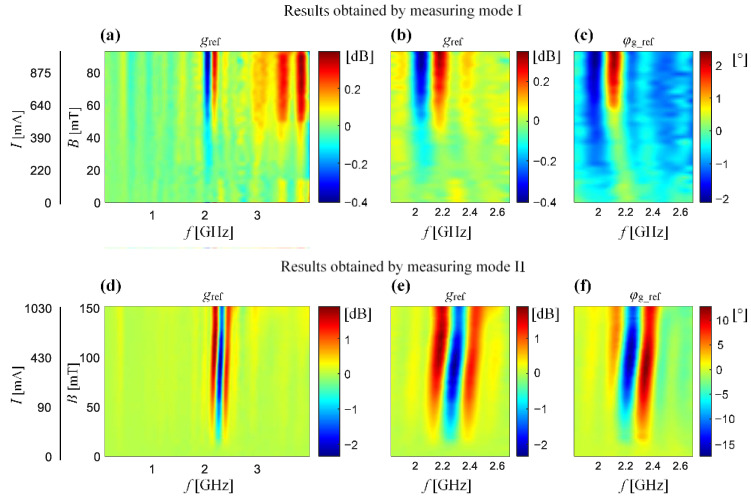
Results of the relative value of the gain factor distribution obtained for the various DC magnetic field strength and the CPW placed in measuring mode I (**a**–**c**) and II (**d**–**f**): (**a**,**d**) the amplitude *g*_ref_ in full frequency range; (**b**,**e**) the amplitude *g*_ref_ in the selected frequency range of interest, (**c**,**f**) the phase *φ*_g_ref_ in the selected the frequency range of interest.

The obtained results of the relative gain factor distribution as a function of the static magnetizing field level *B* and the frequency *f* of the high-frequency component are shown in Figure 7. The first column (Figure 7a,d) shows the distribution of the *g*_ref_ over the entire frequency range. Based on the qualitative analysis of the results, it can be noticed that the greatest changes in the distribution are visible in the frequency range from 1.8 GHz to 2.7 GHz within the applied field level. Therefore, a further analysis was performed only for the mentioned frequency range. When analysing the amplitude (Figure 7b,e) and phase (Figure 7c,f) distributions of the relative gain factor, a clear difference in the intensity of the observed phenomenon can be noticed. The measurement mode II results in a much smaller air gap in the magnetic circuit of the unit: core—ferrite supports—air—sample. This means that the total reluctance in the circuit is lower and, for the same magnetomotive force generated by the magnetizing coils, the value of the obtained field is also higher. Finally, more favourable conditions for the observation of the FMR phenomenon are obtained, even though that this orientation of the sample in the external field causes a much higher value of the demagnetization energy (higher influence of shape anisotropy) [2,19]. At the same time, one can notice a clear shift of the characteristics towards lower frequency ranges for the first CPW orientation mode (measuring mode I). Nevertheless, referring to the aim of obtaining the most favourable measurement conditions, enabling the observation of the phenomenon in a wider dynamic range of changes, further studies were carried out only using the second mode of the CPW orientation.

**Figure 8:**
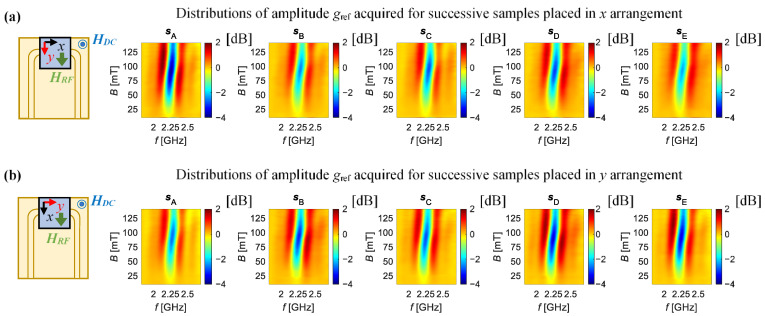
Distributions of the relative gain factor amplitude *g*_ref_ obtained for successive examined samples: when *x*-direction (**a**) and *y*-direction (**b**) of the sample is along the active part of the CPW; s_A_—reference sample; *s*_B_, s_C_, *s*_D_ and *s*_E_ strained samples.

**Figure 9:**
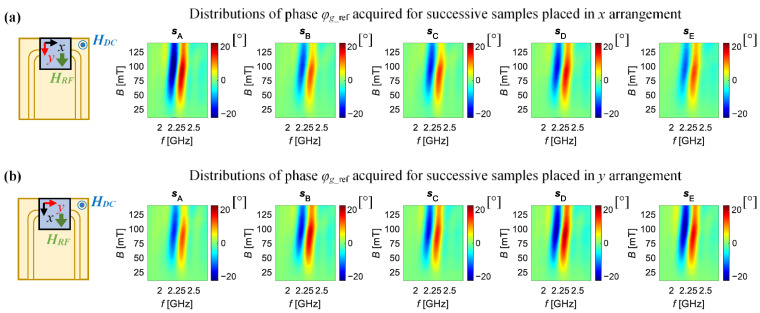
Distributions of the relative gain factor phase *φ*_g_ref_ obtained for successive examined samples: when *x*-direction (**a**) and *y*-direction (**b**) of the sample is along the active part of the CPW; *s*_A_—reference sample; *s*_B_, *s*_C_, *s*_D_ and *s*_E_ strained samples.

**Figure 10:**
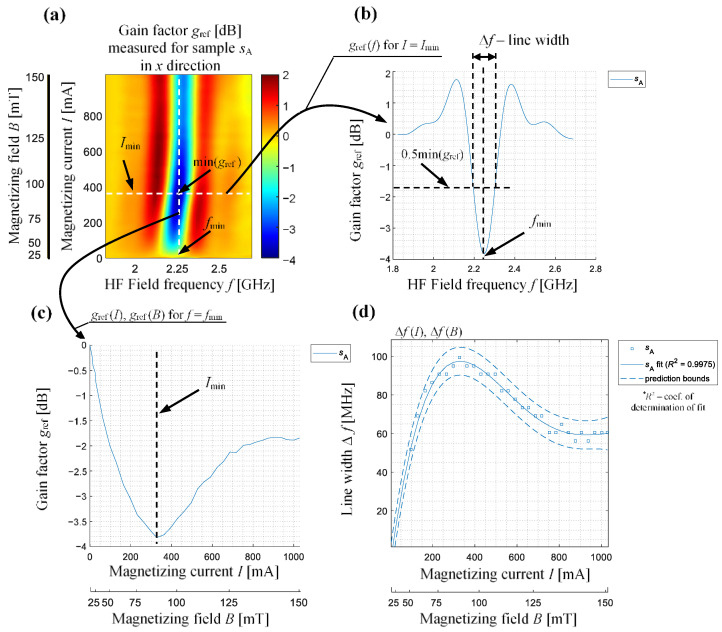
Visualization of the features calculation procedure depicted on the amplitude of relative gain factor *g*_ref_ distribution for sample *s*_A_: (**a**) amplitude distribution of relative gain factor *g*_ref_ as a function of magnetic field components with depicted lines (white dashed lines) localizing its minimum value, (**b**) amplitude of relative *g*_ref_ distribution as a function of frequency *f* of *H*_RF_ component for static field *H*_DC_ conditions for which the minimum of *g*_ref_ occurred (for *I* = *I*_min_ or respectively *B* = *B*_min_), (**c**) amplitude of relative *g*_ref_ distribution as a function of static field *H*_DC_ for frequency *f* of *H*_RF_ for which the minimum of *g*_ref_ occurred, (**d**) the line width Δ*f* changes occurring with static magnetic field conditions.

Figure 8 and Figure 9 show respectively the distributions of the amplitude and the phase of the relative gain factor *g*_ref,_ for two measurement directions. The top rows (Figure 8a and Figure 9a) refer to measurements taken when the samples’ *x*-direction (the tensile direction) was aligned along the active part of the CPW (this setting is called the *x* arrangement), while the bottom rows (Figure 8b and Figure 9b) presents the results when the *y*-direction of the samples was oriented in the same way (ascribed: *y* arrangement). The distributions are presented in a common scale so that all can be compared qualitatively. In the case of the results obtained for the *x* arrangement (Figure 8a and Figure 9a), one can notice that the lowest level of minimum value of *g*_ref_ is obtained for sample s_A_ (the reference sample), as the higher intensity of the blue colour can be observed. One can notice that the level of the *g*_ref_ amplitude’s minimum is generally higher and the distributions are flatter for deformed samples in comparison to the reference one. Similar observations were made for the phase distributions (lower phase variations for deformed samples). On the other hand, the opposite trends of changes occurring with growing strain level in the sample were noticed for the *y* arrangement of samples (Figure 8b and Figure 9b). In this case, the highest level of *g*_ref_ amplitude’s minimum value, as well as the narrowest range of its phase changes, were obtained for the reference sample (the lowest intensity of the obtained distributions). However, in each of these cases, it can be seen that the extreme amplitude and the phase values are obtained for a field of about 100 mT—which is close to current of 400 mA (Figure 10a), and with a further increase of the current value, the intensity of the phenomenon clearly decreases.

**Figure 11:**
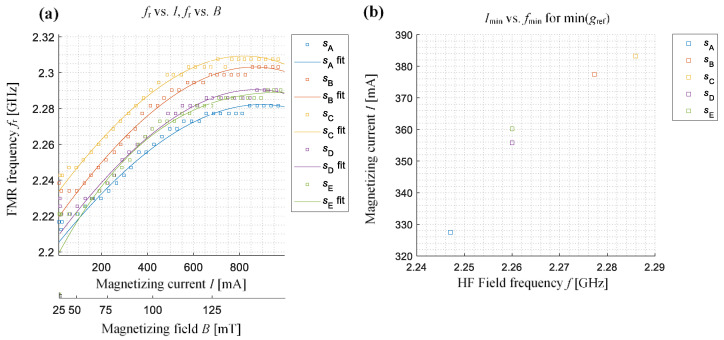
The characteristics of the resonance frequency *f*_r_ versus the magnetizing current (and magnetic field) for different samples aligned with *x*-direction along the active part of CPW: (**a**) changes of the resonance frequency *f*_r_ with an increase of the magnetizing current level *I* and magnetic field level *B*, (**b**) the indication of resonance frequency *f*_min_ and current level *I*_min_ for which the minimum amplitude of relative gain factor was obtained on the distribution.

**Figure 12:**
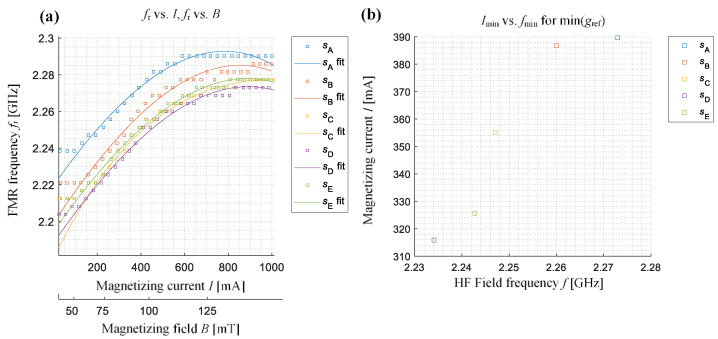
The characteristics of the resonance frequency *f*_r_ versus the magnetizing current (and magnetic field) for different samples aligned with *y*-direction along the active part of CPW: (**a**) changes of the resonance frequency *f*_r_ with an increase of the magnetizing current level *I* and magnetic field level *B*, (**b**) the indication of resonance frequency *f*_min_ and current level *I*_min_ for which the minimum amplitude of relative gain factor was obtained on the distribution.

To confirm the above observations, an analysis was made of all the characteristics features enabling a quantitative evaluation of the achieved characteristics. The definitions and selected distributions of features are presented on the exemplary results achieved for the sample *s*_A_ (placed in *x* arrangement) in Figure 10. The global minimum of the amplitude value of the *g*_ref_, its position on the frequency (ascribed as *f*_min_) and the current (ascribed as *I*_min_) axes, as well as changes in the value of the linewidth Δ*f* (corresponding to the full width at half module band) or the frequency of the minimum value of the *g*_ref_ (pointing the resonance frequency *f*_r_) for different values of the magnetizing field, were calculated. Figure 11 and Figure 12 show the relationship between the resonance frequency *f*_r_ and the of the magnetizing current (or magnetizing field) level and the variation of *g*_ref_ minimum’s location on the (*f*, *I*) plane for all five samples and both samples arrangements (the presented in Figure 11b and Figure 12b current range refers to the magnetic field range of around 10 mT). The obtained characteristics confirm the observations made based on the results presented in Figure 8 and Figure 9. For the *x* arrangement, there is a clear shift of resonance frequency towards higher frequencies for deformed samples, and these differences increase even with the increase in the value of the magnetizing field (or current). Considering the results presented in Figure 11b one can also distinguish three groups of results denoting the position of the resonance on the (*f*, *I*) plane for different samples. The lowest values of current and frequency were obtained for the non-deformed sample *s*_A_. Along with the increase of the deformation level, an increase in both current and frequency value can be seen. A similar relationship, but this time having decreasing character, can be noticed for the second case, i.e., the samples’ *y* arrangement (see Figure 12b). Again, it is possible to distinguish between different samples’ deformation level.

Taking into account the obtained distributions of the location of the global minimum values of the *g*_ref_ factor in the (*f*, *I*) plane along with the strain applied, a change in the relationship between the results obtained for both arrangements can be noticed. It should be remembered that the samples were made of cold-rolled low-carbon film. The rolling direction of the used steel sheet was consistent with the *y*-direction of the samples. In the cold rolling process (inhomogeneous deformation) the rolling magnetic anisotropy (RMA) is introduced in the material [2,5,9]. As the effect of RMA, the easy and hard magnetization axis occur. On the other hand, subsequent samples were subjected to uniaxial stress (homogeneous deformation) in the *x*-direction of the samples. This process also leads to changes in the magnetic properties of the material [2]. Thus, in response to the deformation process, the resultant angular magnetic characteristic further changes. The results of the distributions of the position of the minimum *g*_ref_ factor in plane (*f*, *I*) confirm the occurrence of anisotropy in the tested samples. It should be noted that the samples of the square shape were used for the tests, having the same dimensions in the major axes. This allowed us to minimize the influence of the shape anisotropy on the observed changes in the measured resonance characteristics. In the case of the reference sample, the minimum value of the *g*_ref_ factor occurred at the lower value of the magnetizing current (and thus the *H*_DC_ field) and for the lower driving frequency *f* when the *x*-direction of the sample was consistent with the axis of the active part of the CPW (*y*-direction of the sample parallel to the direction of the *H*_RF_ field). A different relationship can be seen for all four samples subjected to tension. In these cases, the position of *g*_ref_ global minimum for lower magnetizing current (and thus the *H*_DC_ field) and driving frequency *f* was obtained when the samples were oriented to have the *x*-direction along the *H*_RF_ field. The observed change of character can be explained by the change of the angular characteristics and the new location of the resultant axis of easy and hard magnetization as an outcome of plastic deformations introduced to the samples.

## 4. Conclusions

The FMR technique provides the possibility to sense the changes in magnetization dynamics being affected by the destruction process in steels. Additionally, considering that the high-frequency field (due to the skin effect) have the greatest effect on the surface layers of tested materials, the FMR-based techniques can provide important information on the advancement of deformations taking place in the steel elements and can supplement the commonly used methods. Therefore, this work aimed to present the proposed design and then verify the operation of the system based on the VNA-FMR technique for the observation of changes in the dynamics of magnetization of low-carbon steel elements subjected to deformation. The experiments were verified on samples having limited sizes. However, the advantage of the proposed solution of the magnetizing unit and the specially designed CPW can be characterized by the ability to conduct measurements locally anywhere on elements. In the future, the presented system may allow one to increase the mobility of the measurement procedure. The applied solution based on VNA, CPW and a properly designed magnetization system enable the observation of the phenomenon under various conditions of the static and high-frequency field components. The microwave sensor units, such as that basing on a CPW, make it possible to significantly miniaturize them and equip them with subsystems allowing remote operation. That would allow them to be placed on the tested structure. Another possibility of measuring sensitivity enhancement is an application of metasurfaces [36] in the microwave sensing element. This may locally increase magnetic field intensity *H*_RF_.

The results obtained during the experiments confirm the possibility of using this technique to analyse the influence of deformation in magnetic materials. However, it should be emphasized, that the performed works focused solely on the system integration and verification of the general possibility of observing changes in the absorption line as a result of deformations. The research carried out in this work is the first stage of a long process, aimed at developing a measurement methodology in a possibly simplified version and the first verification of the sensitivity scale to changes due to deformation. More detailed conclusions and interpretation of the results require further work, in which the FMR measurements will be combined with a broader material analysis. It must be emphasised that the nature of the changes taking place along with the growth of deformation does not have to be uniform and results, among others, from the value and sign of the magnetostriction coefficient, and consequently from the magnetic permeability and the magnetization value. Their nature is influenced by the level and scope of dislocations occurring in the material. Therefore, the authors are currently preparing further experiments, which will then be carried out. They obey not only the observation of changes in the resonance characteristics for materials at various stages of the straining process, ranging from elastic strains to plastic deformations. The research will also include the observation of microstructural changes. This will enable a broader interpretation of the observed trends in changes in the resonance characteristics, and the results will be presented in the future publication.

## Figures and Tables

**Table 1:** The chemical composition of utilized DC04 film of low-carbon cold-rolled mild steel fulfilling the EN10139 standard.

C	P	S	Mn	Fe
0.08	0.03	0.03	0.4	as remainder

**Table 2:** The basic mechanical properties of the DC04 steel film.

Young Module E [GPa]	Tensile Strength [MPa]	Elongation at Break [%]
180	650	0.8

## Data Availability

Not applicable.

## References

[1] Jiles D.C. (2000). Dynamics of Domain Magnetization and the Barkhausen Effect. Czech. J. Phys..

[2] Cullity B.D., Graham C.D. (2009). Introduction to Magnetic Materials.

[3] Yamazaki T., Furuya Y., Nakao W. (2019). Experimental Evaluation of Domain Wall Dynamics by Barkhausen Noise Analysis in Fe30Co70 Magnetostrictive Alloy Wire. J. Magn. Magn. Mater..

[4] Ducharne B., Le M.-Q., Sebald G., Cottinet P., Guyomar D., Hebrard Y. Characterization and Modeling of Magnetic Domain Wall Dynamics Using Reconstituted Hysteresis Loops from Barkhausen Noise. Proceedings of the First World Congress on Condition Monitoring.

[5] Maciusowicz M., Psuj G. (2020). Time-Frequency Analysis of Barkhausen Noise for the Needs of Anisotropy Evaluation of Grain-Oriented Steels. Sensors.

[6] Hubert A., Schäfer R. (1998). Magnetic Domains: The Analysis of Magnetic Microstructures.

[7] Bilzer C. (2007). Microwave Susceptibility of Thin Ferromagnetic Films: Metrology and Insight into Magnetization Dynamics. Ph.D. Theses.

[8] Kittel C. (2005). Introduction to Solid State Physics.

[9] Morrish A.H. (2001). The Physical Principles of Magnetism.

[10] Neudecker I., Woltersdorf G., Heinrich B., Okuno T., Gubbiotti G., Back C.H. (2006). Comparison of Frequency, Field, and Time Domain Ferromagnetic Resonance Methods. J. Magn. Magn. Mater..

[11] Kalarickal S.S., Krivosik P., Wu M., Patton C.E., Schneider M.L., Kabos P., Silva T.J., Nibarger J.P. (2006). Ferromagnetic Resonance Linewidth in Metallic Thin Films: Comparison of Measurement Methods. J. Appl. Phys..

[12] Lo C.-K., Yaln O. (2013). Instrumentation for Ferromagnetic Resonance Spectrometer. Ferromagnetic Resonance-Theory and Applications.

[13] Chumak O. (2019). Magnetoelastic Properties, Magnetic Anisotropy and Magnetic Damping of Co2YZ Heusler Alloy Thin Films. Ph.D. Thesis.

[14] Maksymov I.S., Kostylev M. (2015). Broadband Stripline Ferromagnetic Resonance Spectroscopy of Ferromagnetic Films, Multilayers and Nanostructures. Phys. E Low-Dimens. Syst. Nanostruct..

[15] Álvarez N., Alejandro G., Gómez J., Goovaerts E., Butera A. (2013). Relaxation Dynamics of Ferromagnetic FePt Thin Films in a Broad Frequency Range. J. Phys. Appl. Phys..

[16] Dubowik J., Glowinski H. (2010). Broad-Band Ferromagnetic Resonance in Thin Magnetic Films and Nanostructures. Curr. Top. Biophys..

[17] Samuel H. (2018). Frequency-Swept Ferromagnetic Resonance Characterization of Permalloy Thin Films.

[18] Zhu B., Lo C.C.H., Lee S.J., Jiles D.C. (2001). Micromagnetic Modeling of the Effects of Stress on Magnetic Properties. J. Appl. Phys..

[19] Kittel C. (1951). Ferromagnetic Resonance. J. Phys. Radium.

[20] Anders W., Biller E. (1971). Ferromagnetic Resonance Linewidth in Bulk Nickel for Various Angles between the Static Magnetic Field and the Surface of the Sample. J. Phys. Colloq..

[21] Schlömann E. (1959). Ferromagnetic Resonance in Polycrystals. J. Phys. Radium.

[22] Tufescu F.M., Óvári T.A., Chiriac H., Stancu A., Cuza A.I. (2003). Stress and temperature effect on the FMR response of nearly zero magnetostrictive amorphous microwires. J. Optoelectron. Adv. Mater..

[23] Ben Hamida A., Sievers S., Pierz K., Schumacher H.W. (2013). Broadband Ferromagnetic Resonance Characterization of GaMnAs Thin Films. J. Appl. Phys..

[24] Guskos N., Glenis S., Zolnierkiewicz G., Typek J., Sibera D., Narkiewicz U. (2011). FMR Study of Temperature Dependence of Magnetic Properties of Nanocrystalline 0.90 (Fe_2_O_3_)/0.10ZnO. Acta Phys. Pol. A.

[25] Merabtine S., Zighem F., Garcia-Sanchez A., Gunasekaran V., Belmeguenai M., Zhou X., Lupo P., Adeyeye A.O., Faurie D. (2018). Origin of Relationship between Ferromagnetic Response and Damage in Stretched Systems. Sci. Rep..

[26] Finkel P., Lofland S. (2007). Stress Dependence and Effect of Plastic Deformation on Magnetic Hysteresis and Anhysteretic Magnetization of FeNi32% Films. J. Appl. Phys..

[27] Dubowik J., Załęski K., Glowinski H., Gościańska I. (2011). Angular Dependence of Ferromagnetic Resonance Linewidth in Thin Films. Phys. Rev. B.

[28] Smokotin E.M., Gusyatskii G.F., Protopopova L.M., Kapitonov A.M. (1978). The Effect of Inhomogeneous Stress on the FMR in Ferrites. Phys. Status Solidi A.

[29] Sahu B.N., Venkataramani N., Prasad S., Krishnan R. (2017). Effect of Thickness on Magnetic and Microwave Properties of RF-Sputtered Zn-Ferrite Thin Films. AIP Adv..

[30] Sievers S., Liebing N., Nass P., Serrano-Guisan S., Pasquale M., Schumacher H.W. (2013). Towards Wafer Scale Inductive Determination of Magnetostatic and Dynamic Parameters of Magnetic Thin Films and Multilayers. IEEE Trans. Magn..

[31] Liebing N., Serrano-Guisan S., Caprile A., Olivetti E.S., Celegato F., Pasquale M., Müller A., Schumacher H.W. (2011). Influence of Sample Geometry on Inductive Damping Measurement Methods. IEEE Trans. Magn..

[32] Noh S., Monma D., Miyake K., Doi M., Sahashi M. (2011). Damping Constant Influence on Spin Dynamics in Field Generating Layer of STO for MAMR Writing Head. J. Phys. Conf. Ser..

[33] PocketVNA User Manual. http://pocketvna.com/styled.

[34] Simons R.N. (2004). Coplanar Waveguide Circuits, Components, and Systems.

[35] Frei W. Modeling of Coplanar Waveguides. https://www.comsol.com/blogs/modeling-coplanar-waveguides/.

[36] Lopato P., Herbko M. (2020). Evaluation of Selected Metasurfaces’ Sensitivity to Planar Geometry Distortions. Appl. Sci..

